# Transcriptomic Analysis of American Ginseng Seeds during the Dormancy Release Process by RNA-Seq

**DOI:** 10.1371/journal.pone.0118558

**Published:** 2015-03-19

**Authors:** Jianjun Qi, Peng Sun, Dengqun Liao, Tongyu Sun, Juan Zhu, Xianen Li

**Affiliations:** Institute of Medicinal Plants Development, Peking Union Medical College, Chinese Academy of Medical Sciences, Beijing, People’s Republic of China; McGill University, CANADA

## Abstract

American ginseng (*Panax quinquefolius* L.) is an important herb that is cultivated in China, North American, and South Korea. It is propagated from seed, but the seed has deep dormancy characteristics described as morphophysiological dormancy. Two-stage temperature stratification, a warm (15–20°C) and cold (2°C) stratification period of 6 months, has been used successfully for seed dormancy release. However, little is known about the molecular mechanisms of seed dormancy release in the stratification process. In this study, seed development after pollination and seed development in the dormancy release process were investigated in American ginseng. The transcriptome during seed dormancy release was analyzed using RNA-Seq technology and 78,207 unigenes (mean length 531 bp) were generated. Based on similarity searches of public databases, 54,292 of the unigenes (69.4%) were functionally annotated. Further, three digital gene expression (DGE) libraries were sequenced and differences in gene expression at three stages during seed cold stratification were examined. The greatest number of differentially expressed genes occurred in the 90DCS versus 180DCS libraries, while the lowest number of differentially expressed genes occurred in the 135DCS verus 180DCS libraries. GO enrichment analysis revealed that 59, 29, and 39 GO terms were significantly enriched in the biological process, molecular function, and cell component GO categories, respectively. There were 25,190 genes with KEGG pathway annotation in the three DGE libraries and their enrichment pathways were compared. The gene expressions of 30 selected unigenes were validated using quantitative PCR. This study is the first to provide the transcriptome sequences for seed dormancy release in American ginseng, and demonstrates the successful use of DGE profiling data for analyzing transcriptomic variation during dormancy release. These data provide a basis for future researches of seed dormancy in morphophysiological dormancy seeds in non-model plants.

## Introduction

American ginseng (*Panax quinquefolius* L.), one of the most widely used medicinal plants of the Araliaceae family, has been introduced and cultivated in China for over forty years. It is propagated from seed. The embryo in the newly harvested seed is not fully developed (average length 0.5mm), and needs a warm (15–20°C) and cold (2°C) stratification period of 6 to 18 months [1, 2, 3]. Based on the Baskin and Baskin classification theory of seed dormancy [4], ginseng seeds belong to the morphophysiological dormancy (MPD) class, which describes a combination of morphological and physiological dormancy. Many factors affect MPD seed germination, including the stratification period, seeding time and depth, temperature, and spacing [1, 5]. At present, two methods have been used for seed treatment. One is a conventional method in which the newly harvested seeds are buried in sand outdoors for 18 to 22 months under seasonal changes of temperature. The other method is a two-stage process: a warm stratification stage during which the embryo begins to grow, and a cold stage during which the embryo completes its development and the endogenous dormancy of the seed is overcome. Thomas et al. suggested that it was possible to induce germination by manipulating ginseng seeds through three months each of warm (15°C) and cold (2°C) temperatures [1]. So far, although the two-stage ginseng seed stratification technology has been widely used [1, 6, 7], the internal molecular mechanism of seed dormancy release is still unclear.

Mining the functional genes, especially those that encode the key enzymes involved in ginsenoside biosynthesis in American ginseng has become a focus of attention in recent years. Chen et al. [8] have generated ESTs (expressed sequence tags) that will be useful for studying functional genomics in American ginseng, and which can be applied to molecular modification of the ginsenoside biosynthetic pathway. Searching public databases is another method that has been used to identify functional genes for cloning [9]. Over the last few years, high-throughput sequencing, such as 454-sequencing and RNA-sequencing (RNA-Seq), has provided new strategies for analyzing the functional complexity of transcriptomes and the sequencing data that are now available have opened up opportunities for mining functional genes in non-model plants such as American ginseng. Sun et al. and Wu et al. obtained many unigenes by 454 sequencing and identified all the known enzymes involved in ginsenoside backbone synthesis [10–12]. Qi et al. analyzed the transcriptome data obtained using 454-sequencing in a type of MPD Paris *polyphylla* seeds [13]. RNA-Seq technology based on pyrosequencing is a popular and powerful tool for species that lack reference genome information. RNA-Seq is less costly, more efficient, and allows faster gene discovery and more sensitive and accurate profiles of the transcriptome than microarray analysis or other techniques [13–18]. To better understand the molecular mechanisms of seed dormancy in ginseng seeds, we used RNA-Seq technology to identify and characterize the expression of a large number of genes, especially those differentially expressed during dormancy release.

The aim of this work was to gain an understanding of the molecular mechanisms associated with seed dormancy release in American ginseng and establish a sound foundation for future molecular studies. For seed transcriptome sequencing, four seed samples were taken to form a sequencing library, two samples at warm stratification (no split and split seeds) and two samples at cold stratification (135 days and 180 days during the cold treatment). For the digital gene expression (DGE) analysis, three seed samples were taken at 90 days (90DAS), 135 days (135DAS) and 180 days (180DAS) after the warm stratification. We sequenced cDNA libraries from American ginseng seed during the dormancy release process and analyzed the expression of different genes in seeds during cold stratification to identify dormancy-related genes with the aim of constructing a database for this species. To our knowledge, this is the first report on the transcriptome profiling of American ginseng seed dormancy using RNA-Seq technology.

## Methods

### Plant materials

American ginseng (*Panax quinquefolius* L.) was cultivated in an experimental field in the Institute of Medicinal Plant Development, Beijing, China. The flowers were labeled to make it easy to calculate the date after pollination. After pollination, the ovules used to observe embryo development were sampled every seven days for a total of eleven times, therefore, sampling lasted for 77 days. The ovules were fixed using FAA (95% alcohol: acetic acid: formalin: water = 10:1:2:7) and made into tablets for microscopic inspection. Two-stage seed treatment method was employed for dormancy release. American ginseng seeds were stratified for 3 months in a sandbox at a warm temperature (15°C) during which time most of the seeds completed their anatomical development (split seeds), and then cold (2°C) stratification was applied for another 3 months for physiological dormancy release. For transcriptome sequencing, four seed samples were taken at two seed treatment periods, that is, two samples at warm stratification (no split and split seeds) and two samples on Day 135 and 180 during the cold stratification. For the digital gene expression (DGE) analysis, three seed samples were taken separately on Day90, Day 135 and Day 180 during the cold stratification.

### RNA extraction, library preparation, and RNA-seq

The seeds were sampled from three biological replications at each stage to produce an independent pool. Total RNA was extracted from seed samples using an RNA Extract kit (BioTeke, Beijing, China). To construct the transcriptome library, the RNA from the four seed samples described above was pooled by mixing in equal quantities. The three DGE libraries consisted of separate RNA extracts from the three cold stratification samples. Each library was pooled by mixing equal quantities of RNA from the three biological replications for each stage. Each pool was sequenced once because RNA-seq data are highly replicable with relatively little technical variation. RNA integrity was confirmed using a 2100 Bioanalyzer (Agilent Technologies Santa Clara, CA, USA). Oligo-(dT) magnetic beads were used to isolate poly-(A) mRNA from total RNA, and mRNA was fragmented in fragmentation buffer. Using these short fragments (200 bp) as templates, random hexamer primers were used to synthesize first-strand cDNA. Second-strand cDNA was synthesized using buffer, dNTPs, RNaseH, and DNA polymerase I. Short double-stranded cDNA fragments were purified with a QiaQuick PCR extraction kit (Qiagen, Venlo, The Netherlands), resolved with EB buffer for end reparation and adding poly (A), then ligated to sequencing adapters. After purification via agarose gel electrophoresis, suitable fragments were enriched by PCR amplification before library sequencing on an Illumina HiSeq 2000 platform (San Diego, CA, USA).

### De novo assembly and function annotation

The raw reads were saved in fastq format, and the dirty raw reads were removed prior to analyzing the data. Three criteria were used to filter out dirty raw reads: adaptors, more than 5% “N” bases, low-quality reads with more than 20% of the bases having a quality value ≤ 10. All subsequent analyses were based on clean reads. De novo assembly of the clean reads was performed using Trinity (release 20110713) [19]. The command-line parameters were as follows:—seqType fq,—min_contig_length 100,—min_glue 3,—group paris_distance 250,—path_reinforcement_distance 85, and—min_kmer_cov 2. In this paper, the assembled reads are referred to as unigenes. All non-redundant transcripts were used in searches against the NR, Swiss-Prot, KEGG, and COG databases using the BLASTALL package with the significant threshold E-value ≤ 10^−5^. The annotation for each known gene from the best BLASTX hit was parsed and assigned to the corresponding transcript. For transcripts with no hits to any of the searched databases, ESTScan [20] was used to predict the coding region and decide the sequence direction. Blast2GO [21] and WEGO [22] were used to assign GO annotations to the transcripts for functional classification.

### DGE library preparation, sequencing, and bioinformatics analysis

Total RNA \from each of the three cold stratification seed stages, 90DAS, 135DAS, and 180DAS, were used to construct three tag libraries. The libraries were prepared in parallel for DGE sequencing using the Digital Gene Expression Tag Profile Kit (Illumina). The detailed process was as described by Meng et al. [23]. Reads with adaptors, reads in which unknown bases were more than 10%, and low quality reads (percentage of the low quality bases (quality value <5) was more than 50% in one read) were removed. The clean reads were mapped to the transcriptome unigene sequences using the SOAPaligner/soap2 (version 2.20) alignment program, allowing a maximum of 2-bp mismatches per read. To estimate the expression levels of the transcripts, the number of uniquely mapped reads for each unigene for the three cold treatment stages was competed and normalized to RPKM values (reads per kilobase per million mapped reads) using the RPKM formula described by Mortazavi et al. [24]. The longest mapped read was used to calculate the expression level and coverage when multiple unigenes represented one gene. For genes with missing values in a specific sample the RPKMs were adjusted to 0.001. The RPKM values were then compared pairwise as: 90DAS/135DAS, 90DAS/180DAS, and 135DAS/180DAS. Enriched P-values were calculated using the formula described by Audic and Claverie [15], and Liu et al. [16]. The P value was used to identify genes expressed differentially between the paired treatments. Significantly DGEs were identified using a FDR (false discovery rate) threshold of ≤ 0.001 and a minimum two-fold change. Based on log2-transformed RPKM values, the expression patterns of 22,832 DGEs were clustered in Genesis (1.7.6) (http://genome.tugraz.at) using the K-means algorithm and Pearson’s correlation distance.

### qPCR analysis

Total RNA was isolated from seed, embryo, endosperm, root, stem, and leaf of American ginseng using the RNeasy Plant Kit (BioTeke). Approximately 1 μg of DNase I-treated total RNA from each tissue was converted into single-stranded cDNA using a PrimeScript RT reagent Kit (Takara, Dalian, China). The cDNA products were then diluted 10-fold with deionized water before use as templates for RT-PCR.

The RT-PCR analyses were performed three times with independent RNA samples. Primers for 30 of the transcriptome unigenes were used in the PCR reaction (S1 Table). 18S RNA gene was used as an internal control to assess the differential regulation of the genes. The semi-quantitative PCR reaction was performed in a total volume of 25 μl containing 10 pmol of the gene-specific primers, 20 μM of dNTPs mix, 1.5 mM MgCl2, 1× *Taq* polymerase buffer, and 1 U *Taq* polymerase (Takara, DaLian, China). The thermal cycling conditions were as follows: initial denaturation at 94°C for 5 min, then 35 cycles of denaturation at 94°C for 30 sec, re-annealing at 55°C for 30 sec, and elongation at 72°C for 30 sec. A final elongation was performed at 72°C for 5 min. Amplicons were separated on 2% agarose gel and stained with ethidium bromide and then documented on a gel-imager. For real-time PCR analysis, each reaction contained 10μl 2×SYBR Premix DimerEraser (Takara, Dalian, China), 1μM each of the forward and reverse primers, and 1μl of template cDNA. The total reaction volum was 20μl. PCR amplification was performed under the following condition: 95°C for 30 sec, followed by 40 cycles of 95°C for 5 sec and 60°C for 30 sec, using CFX96^TM^ Real-Time system (Bio-Rad, USA). The specificity of real-time PCR primers was confirmed by melting curve (S1 Table). Relative expression was calculated as 2^-ΔΔCT^ and normalized to that of the actin gene[25]. The real-time PCR analysis were performed three times with independent RNA samples.

## Results and Discussion

### Seed development and stratification

**Fig 1:**
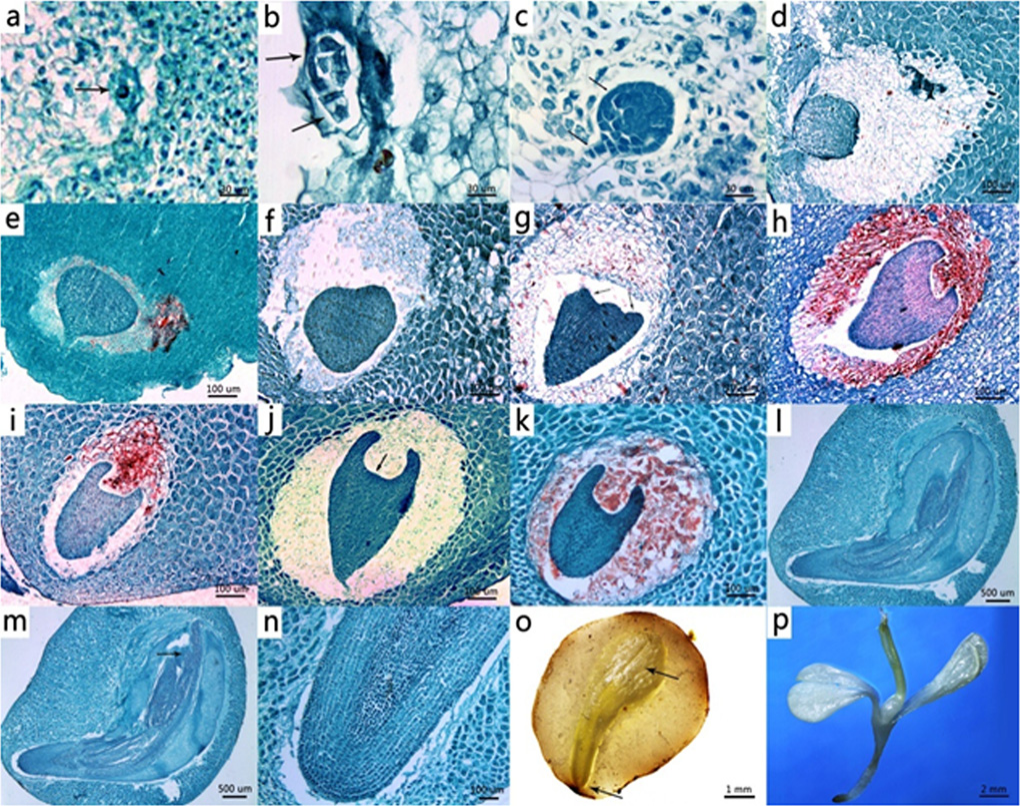
Development and stratification of American ginseng seeds. (a) Zygotic embryo at 7 days after pollination (DAP). (b) Embryo with 16 cells. (c) Globular stage embryo at 28 DAP. (d-e) Late globular or triangular stage embryo at 38 DAP. (f-g) Late triangular or early heart stage embryo at 48 DAP. (h-i) Early torpedo stage embryo at 58 DAP. (j) Late torpedo stage embryo at 63–77 DAP indicated shoot apical meristem. (k) Warm stratified seeds indicated no split; (l-o) split seeds after warm stratification. (p) Seedling after cold stratification.

The *P*. *quinquefolius* zygote was still in a state of rest at 78 days after pollination (DAP). The proembryo was formed gradually after a first asymmetric division at about 10–14 DAP, and the early globular embryo was seen at 21–28 DAP. At 38 DAP, the globular embryo began transfer to the triangular stage embryo and the suspensor began to degenerate; at the same time, the endosperm became fully developed. Then, from 38–58 DAP, the leaf primordium, the heart stage embryo, and the early torpedo stage embryo gradually formed. At the time of harvesting the seeds, the embryos had stopped developing and were in the late torpedo stage at about 68–78 DAP. The brief development stage of an American ginseng seed after pollination is shown in Fig. 1 (a–j). It can be seen that the seed is not fully developed. For anatomical development, the seed needs a warm stratification period after which cotyledons, hypocotyls, radicals, and epicotyls become visible (Fig. 1 (k–o)). As suggested earlier by Thomas et al. [1, 2], we performed a warm stratification of about 3–4 month for morphological dormancy release and then a cold stratification of about 3–6 month to overcome endogenous dormancy.

### RNA-Seq and de novo assembly

**Fig 2:**
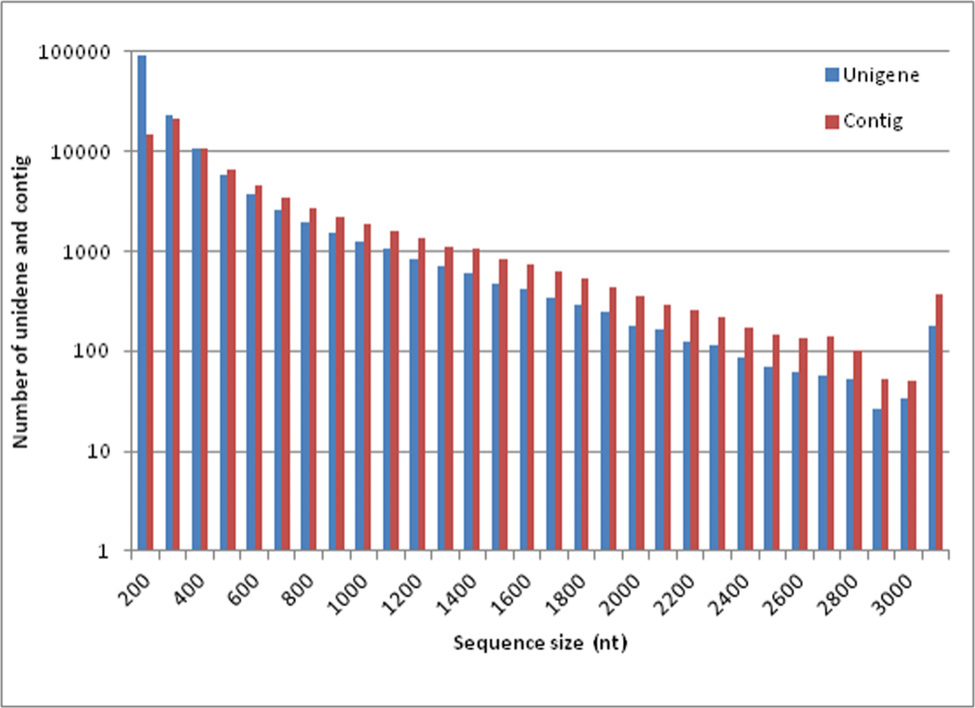
Size distributions of the contigs and unigenes in the American ginseng seed transcriptome.

To obtain an overview of the seed transcriptome during the dormancy release process, a cDNA library was generated from RNA isolated from four seed treatments including anatomical development and physiological after-ripening stages, and paired-end sequenced using the Illumina platform. After cleaning and quality checks, approximately 25.86 million clean reads were assembled into 148,244 contigs (Table 1). The mean contig size was 275 bp. Using paired-end joining and gap-filling, these contig were further assembled into 78,207 unique sequences with a mean size of 531 bp. The size distributions of the contigs and unigenes are shown in Fig. 2. The raw transcriptome reads were deposited in the National Center for Biotechnology Information (NCBI) Short Read Archive (accession number: SRR1586196)

**Table 1:** Summary of the de novo transcriptome assembly of *P*. *quinquefolius* seeds.

Total number of raw reads	27,622,934
Total number of clean reads	25,865,496
Total number of clean nucleotides (nt)	2,327,894,640
Total number of contigs	148,244
Mean length of contigs (nt)	275
Total number of unigenes	78,207
Mean length of unigenes (nt)	531

### Annotation of the *P*. *quinquefolius* seed transcriptome

Approximately 54,292 unique sequences were annotated using BLASTX searches (cut-off E-value 10^−5^) against public databases: NCBI’s non-redundant protein sequence (nr) and nucleotide sequence (nt) databases, Swiss-Prot protein database, Kyoto Encyclopedia of Genes and Genomes (KEGG), Gene Ontology (GO) database, and clusters of orthologous groups (COG). A summary of the transcriptome functional annotation is given in Table 2. A total of 23,915 unigenes (30.6%) did not find matches in any of the databases.

**Table 2:** Annotation of the American ginseng seed transcriptome.

Database	Number of unigene annotated	Percentage (%)
Nr	51,909	66.4
Nt	43,713	55.9
Swiss-Prot	29,786	38.1
KEGG	26,909	34.4
COG	15,552	19.9
GO	22,980	29.4
ALL	54,292	69.4

Among the annotated unigenes, many were predicted to be related closely with seed dormancy release proteins such as: dormancy/auxin associated protein [16], somatic embryogenesis receptor kinase [26], seed maturation protein, abscisic acid (ABA) 8-hydroxylase (CYP707A), 9-cis-epoxycarotenoid dioxygenase (NCED), gibberellin 2-oxidase, gibberellin 3-oxidase, gibberellin 20-oxidase, GRAS-domain transcription factors, DELLA protein, and 1-aminocyclopropane-1-carboxylate synthase (ACC)[27]. Detailed information about selected unigenes that might be associated with seeds dormancy release or seeds development is available in S2 Table. We designed specific primers for qPCR analysis of some of the annotated seed dormant related unigenes.

### Functional classification

**Fig 3:**
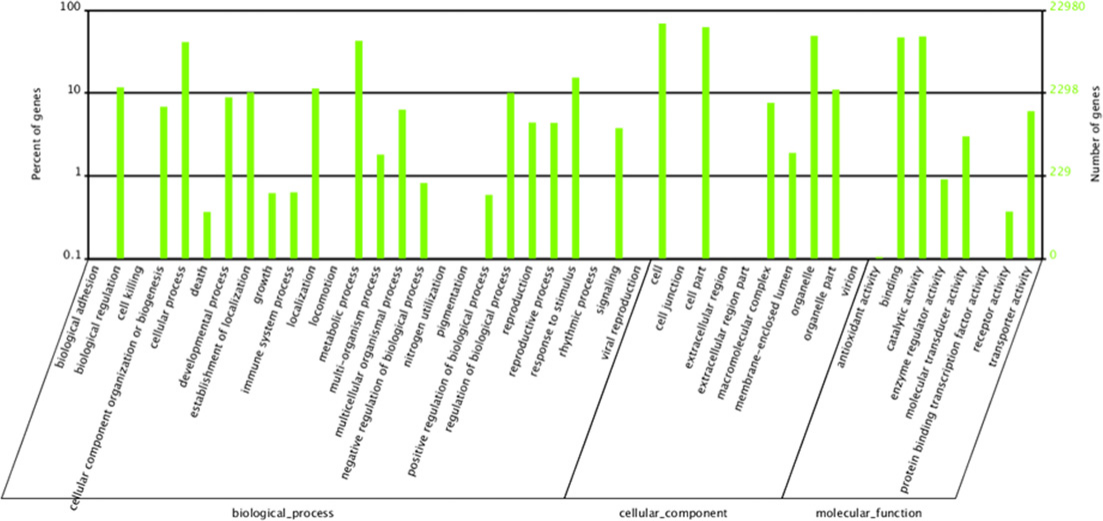
Gene Ontology (GO) categories of the assembled American ginseng unigenes. The unigenes were assigned to the three GO categories: biological process, cellular component, and molecular function.

We used GO, COG, and KEGG assignments to classify the functions of the predicted unigenes. Based on the Arabidopsis Information Resource Gene Ontology Slim classification provided by Blast2GO [28], 22,980 unigenes were categorized into 44 functional groups under the three main GO divisions (biological process, cellular component, and molecular function). In the biological process category, metabolic process (9789, 42.60%), cellular process (9456, 41.15%), and response to stimulus (3524, 15.34%) were the predominant groups (Fig. 3). In the cellular component category, cell (15848, 68.96%) and cell part (14327, 62.34%) were the predominant groups, followed by organelle (11241, 48.92%) (Fig. 3). In the molecular function category, catalytic activity (11048, 48.08%) and binding (10767, 46.85%) were the predominant groups, followed by transporter activity (1386, 6.03%) (Fig. 3).

**Fig 4:**
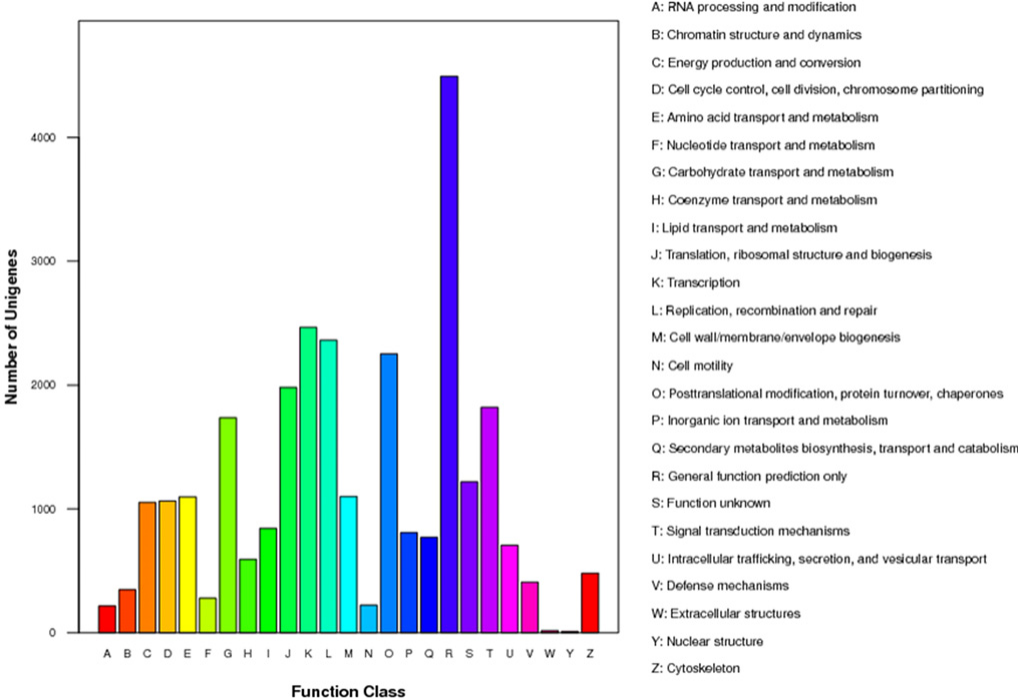
Clusters of orthologous groups (COG) classification of the assembled American ginseng unigenes. A total of 15,552 unigenes were classified into 25 functional categories according to their predicted gene products (COG cut-off E-value was 10–5).

A total of 15552 unigenes were assigned a COG classification. Among the 25 categories, transcription (2464, 15.84%) represented the largest group, and transcripts associated with replication, recombination and repair (2361, 15.18%), posttranslational modification, protein turnover, chaperones (2251, 14.47%), and translation, ribosomal structure and biogenesis (1983, 12.75%) were the next most common groups. The categories extracellular structures (15, 0.09%) and “nuclear structure” (9, 0.06%) represented the smallest groups. However, categories with no concrete assignment, such as function unknown (1219, 7.84%) and general function prediction (4493; 28.89%) accounted for a large proportion of the transcripts (Fig. 4).

KEGG Pathway mapping indicated the possible biological interpretation of the assigned functions. 26,909 unigenes were assigned to 126 pathways in the KEGG database. The most represented pathways included metabolic pathways (6117, 22.73%), biosynthesis of secondary metabolites (2633, 9.78%), and plant hormone signal transduction (1536, 5.71%). In particular, some unigenes that were predicted to be involved in the phytohormone related pathway, such as zeatin biosynthesis (205, 0.76%), carotenoid biosynthesis (154, 0.57%), steroid biosynthesis (85, 0.32%), diterpenoid biosynthesis (79, 0.29%), and brassinosteroid biosynthesis (26, 0.01%) (S3 Table), might be important in seed development and seed dormancy release in American ginseng.

### DGE library sequencing and mapping to the reference transcriptome database

Three seed sample DGE libraries (90DAS, 135DAS and 180DAS) were sequenced using tRNA-Seq and 8,424,608, 10,034,797, and 8,710,395 clean reads, respectively, were obtained after filtering out the raw reads (Table 3). Among them, 76.06%, 75.16%, and 78.60% of the reads in 90DAS, 135DAS, and 180DAS, respectively, mapped to reference genes and more than 50% of the reads had perfect matches and unique matches to reference genes [29] (Table 3). Sequencing quality was evaluated using saturation analysis and randomness assessment (S1 Fig.).

**Table 3:** Summary of read numbers in the RNA-Seq data from American ginseng seeds.

Summary	90DAS	135DAS	180DAS
Total clean reads	8,424,608	10,034,797	8,710,395
Total clean reads/total raw reads (%)	99.25	99.47	99.50
Mapped reads	6,331,657	7,632,437	6,846,348
Mapped reads/total clean reads (%)	76.06	75.16	78.60
Perfect match	4,538,801	5,445,325	4,961,635
Perfect reads/total clean reads (%)	53.88	54.26	56.96
Unique match	4,445,654	5,525,636	5,105,795
Unique match/total clean reads (%)	52.77	55.06	58.62
Unmapped reads	2,092,951	2,402,360	1,864,047
Unmapped reads/total clean reads (%)	24.84	23.94	21.40

90DAS, 135DAS, and 180DAS: three ginseng seed DGEs libraries of 90 days, 135 days, and 180 days after the warm stratification.

For mRNA expression, heterogeneity and redundancy are two significant characteristics. While the majority of mRNAs are expressed at low levels, a small proportion of mRNAs are highly expressed. Therefore, the distribution of unique reads was used to evaluate the normality of the RNA-Seq transcriptome data. As shown in S2 Fig., the distribution of unique reads over the different reads abundance categories showed similar patterns for all three RNA-Seq libraries. The similarity distribution showed that more than 32% of the sequences had a similarity of 80%, while approximately 50% of the hits had a similar range.

### Gene expression profiles among three seed dormancy stages

**Fig 5:**
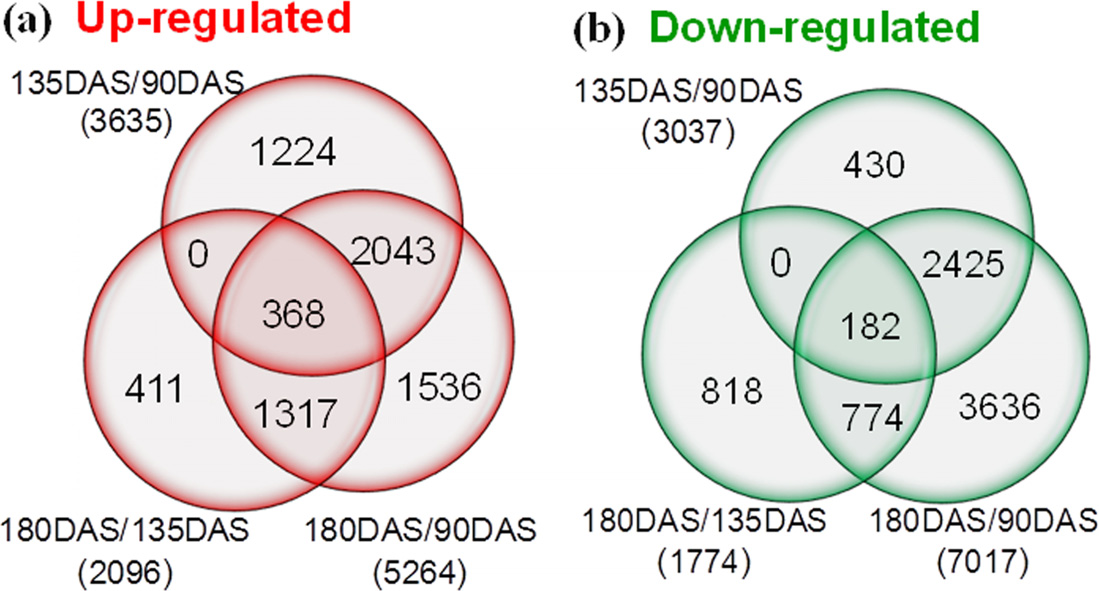
Venn diagram showing number of DEGs revealed by paired comparison (FDR≤0.001, |log2FC|≥1). The numbers of up- and down-regulated genes in comparisons of 135DAS/90DAS, 180DAS/135DAS/, and 180DAS/90DAS libraries. DEGs: Differentially expressed genes.

Differences in gene expression among the three seed dormancy stages during the cold stratification were examined, and differentially expressed genes (DEGs) were identified by the pairwise comparisons: 90DAS-VS-135DAS, 90DAS-VS-180DAS, and 135DAS-VS-180DAS. Fig. 5 (a) and (b) showed the distribution of the total number of DEGs up- or down-regulated between two compared stages and their relationship among the different comparisons. 3635 and 3037 DEGs were found to be up- and down-regulated separately in the 135DAS to 90DAS comparison. A similar number of DEGs were differentially expressed between 180DAS and 90DAS. The comparison of 180DAS and 135DAS identified 2096 enhanced DEGs and 1774 decreased DEGs, which were less than the former two comparisons, indicating less genes showed differential expression through 135DAS to 180DAS. Only the expression abundance of 368 and 182 DEGs were significantly increased or decreased by at least 2 folds throughout the cold stratification, indicating that most transcripts were transiently significantly regulated by the switches of stratification process. For example, of 3635 enhanced Unigenes in 135DAS to 90DAS comparison, 1224 Unigenes were increased only in 135DAS, and 2043 Unigenes were overlapped with those in 180DAS to 90DAS comparison, indicating that they still remained higher expression levels in 180DAS after the increase since 135DAS, but no quantitative difference from 135DAS. These differences in gene expression are consistent with the seed dormancy release process. Some reports have shown that gene activity was lowest in the early stages of dormancy and peaked around the time of dormancy break [24, 30]. In the 90DAS, 135DAS and 180DAS libraries, 21.38%, 17.67%, and 25.58% genes, respectively, found no matches in the nr or GO databases. These genes may be candidates for future investigations.

### Functional classification of DEGs during seed dormancy release

We used GO and KEGG assignments to classify the functions of the DEGs identified in the pairwise comparisons of the 90DAS, 135DAS and 180DAS cDNA libraries during seed dormancy release. Of the assigned GO terms, 59, 29, and 39 GO terms were significantly enriched in the cellular component, molecular function, and biological process GO categories respectively (S4 Table). The significantly enriched GO terms were almost all general cell functions, such as non-membrane-bounded organelle, intracellular organelle part, structural molecule activity, and oxidoreductase; however, the DEGs assigned the steroid binding, steroid dehydrogenase activity, hormone binding, and auxin transmembrane transporter activity GO terms may be important candidate genes for seed dormancy release studies, although these terms were not significantly enriched.

There are 25,190 genes with KEGG pathway annotation in DGE libraries. In the 90DAS/135DAS, 90DAS/180DAS, and 135DAS/180DAS comparisons, 2,864, 4,829, and 1,713 DEGs mapped to 122, 124, and 113 KEGG pathways, respectively. Of the 2,864 DEGs in the 90DAS/135DAS comparison, 2,378 (83.03%) mapped to 31 significantly enriched pathways (p-value <0.05). Of the 4,829 DEGs in the 90DAS/180DAS comparison, 4,117 (85.26%) mapped to 36 significantly enriched pathways (p-value <0.05), and of the 1,713 DEGs in 135DAS/180DAS comparison, 1821 (1.06%) mapped to 34 significantly enriched pathways (p-value <0.05) (S5 Table). Of the genes associated with the significantly enriched KEGG pathways, those involved in steroid biosynthesis, flavonoid biosynthesis, flavone and flavonol biosynthesis, benzoxazinoid biosynthesis, starch and sucrose metabolism, and zeatin biosynthesis might be important candidate genes for American ginseng seeds dormancy release and worthy of further study. Some of the DEGs assigned to pathways that were not significantly enriched, such as indole alkaloid biosynthesis, carotenoid biosynthesis, circadian rhythm, brassinosteroid biosynthesis, and plant hormone signal transduction could also be of interest.

### Expression profiles of GA, ABA metabolism and other related genes

**Fig 6:**
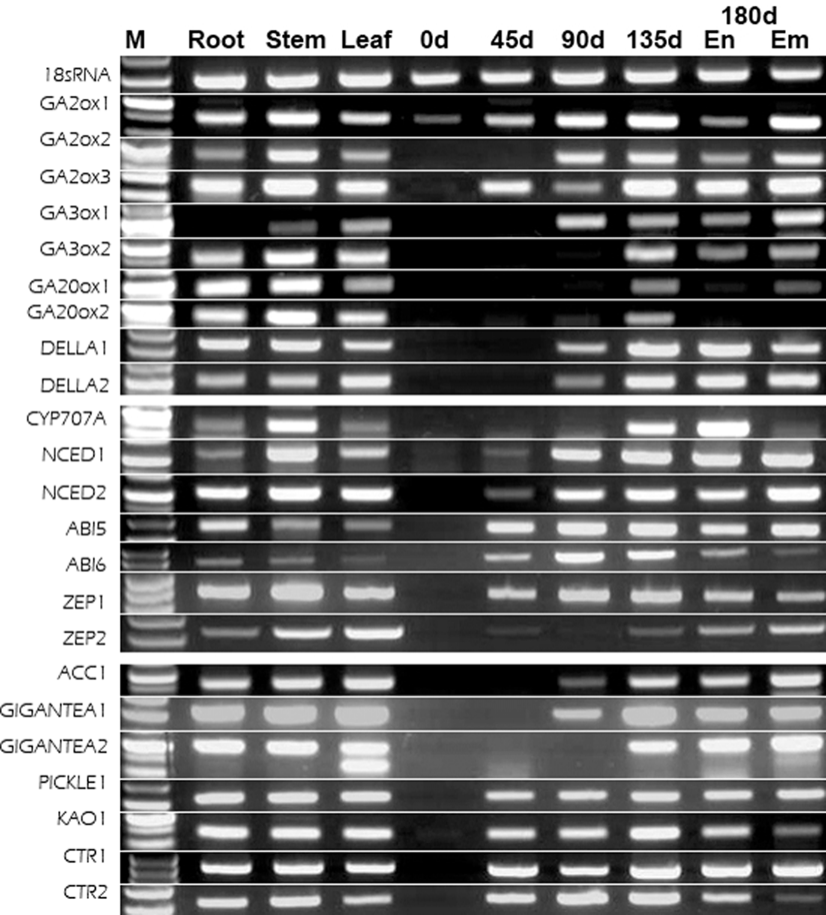
Semi-quantitative PCR analysis of 23 selected genes from the American ginseng seeds libraries. M: maker; 0d, 45d, 90d, 135d, and 180d: seed stratification period; Em: embryo; En: endosperm; root, stem, and leaf as control tissues.

Many reports have shown that stratification can lead to increased expression of the gibberellic acid (GA) biosynthesis genes *GA20ox* and *GA3ox*, but deceased expression of the GA catabolic gene *GA2ox* in Arabidopsis seeds [31, 32]. Additional studies have identified specific genes correlated with dormancy maintenance (*NCED*, *ZEP* (zeaxanthin epoxidase) and ABI (ABA insensitive)) and dormancy release via ABA catabolism (*CYP707A*) [33–35]. In this work, we evaluated the DGE library using 30 unigenes including those that were associated with GA and ABA, as well as *DELLA* (a negative regulator of GA signal), *ACC* (1-aminocyclopropane-1-carboxylate synthase), *GIGATEA*, *PICKLE* (CHD3-type chromatin-remodeling factor PICKLE), *KAO* (Ent-kaurenoic acid oxidase), and *CTR*(serine/threonine-protein kinase CTR1) and others [36–38]. For the semi-quantitative PCR analysis, 41 primers were designed for the 30 unigenes (the primers are listed in S1 Table). Twenty-three of the primers successful amplified 23 of the 30 selected unigenes (Fig. 6). The PCR data for 19 of the 23 amplified (the exceptions were *GA2ox2*, *GA2ox3*, *GA3ox2*, and *GA20ox1*) were basically consistent with the RNA-Seq data for the 90DAS, 135DAS and 180DAS samples (Table 4). We also analyzed the expression levels of these 23 unigenes on Day 0 and 45 of the warm stratification and in three control tissues (root, stem, and leaf). The results showed that almost all unigenes had no or weak expression in the early stage (0 days) of seed stratification.

**Table 4:** Different expression of The RPKM Studied genes in quantitative PCR.

Gene name	GeneID	Description	Length(bp)	Product length(bp)	90DAS (RPKM)	135DAS (RPKM)	180DAS (RPKM)
*GA2ox1*	Unigene3243	gibberellin 2-oxidase	524	147	48.94	29.70	25.42
*GA2ox2*	Unigene12643		1222	166	0.18	-	1.6
*GA2ox3*	Unigene12643		1222	129	0.18	-	1.6
*GA2ox4*	Unigene12642		1322	167	1.02	3.28	3.70
*GA3ox1*	Unigene57244	gibberellin 3-oxidase	1242	159	32.08	150.08	212.25
*GA3ox2*	Unigene64656		1104	156	24.04	8.69	17.38
*GA20ox1*	Unigene60909	gibberellin 20-oxidase	225	169	10.99	8.04	9.57
*GA20ox3*	Unigene61797		1289	168	-	0.14	-
*DELLA3*	Unigene11898	DELLA protein	2615	330	3.7	2.28	2.7
*DELLA7*	Cl477.Contig1		1976	207	107.46	90.3	97.14
*CYP707A*	Unigene23879	ABA 8'-hydroxylase CYP707A	1728	140	2.34	8.17	2.61
*CYP707A4*	Unigene59678				4.33	3.04	13.42
*NCED*	Unigene27579	9-cis-epoxycarotenoid dioxygenase	1988	230	38.92	33.14	32.22
*NCED*	Unigene27579		1988	205	38.92	33.14	32.22
*ABI5–1*	Unigene25542	abscisic acid insensitive 5-like protein	664	250	101.29	76.86	12.09
*ABI5–2*	Unigene25537		1786	413	10.7	4.76	1.75
*ZEP1*	Unigene3202	zeaxanthin epoxidase	873	406	13.66	31.3	41.5
*ZEP2*	Unigene70254		570	338	3.95	11.75	22.68
*ACC1*	Unigene23819	1-aminocyclopropane-1-carboxylate synthase	904	192	46.03	66.46	584.10
*GIGANTEA1*	Unigene11747	*GIGANTEA*	4188	310	83.79	73.76	70.71
*GIGANTEA2*	Unigene13175		348	200	5.17	13.00	12.94
*PICKLE1*	Unigene58801	CHD3-type chromatin-remodeling factor PICKLE	4263	422	20.47	9.17	6.57
*KAO1*	Unigene14671	3-ketoacyl CoA thiolase 1	305	237	2.95	30.26	23.76
*KAO2*	Unigene45375		433	189	1.04	24.66	23.07
*CTR1*	Unigene67197	Serin/threonine protein CTR1-like	876	386	69.59	42.14	16.77
*CTR2*	Unigene67197		876	322	69.59	42.14	16.77
*DRM1*	Unigene26810		963	210	14.24	10.71	27.25
*DRM2*	Unigene944		247	167	1.82	-	6.34

90DAS, 135DAS, and 180DAS: three ginseng seed DGEs libraries of 90 days, 135 days, and 180 days after the warm stratification

**Fig 7:**
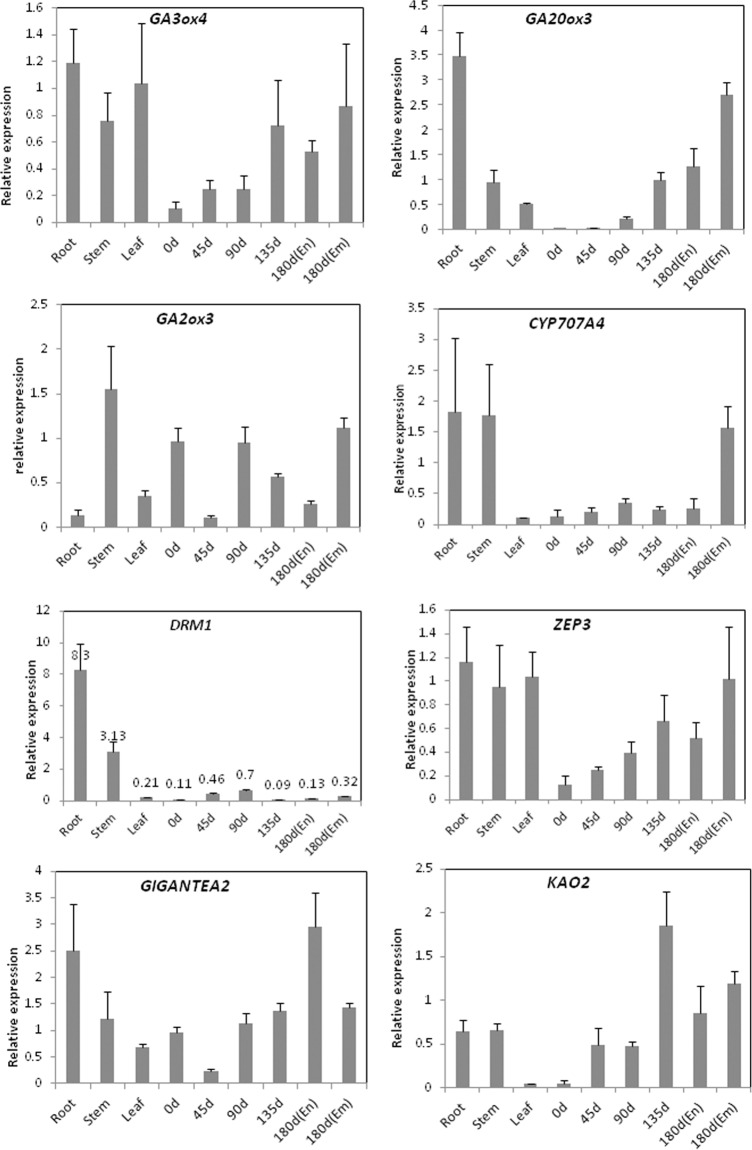
Relative expression level analysis of 8 selected genes. 0d, 45d, 90d, 135d, and 180d: cold seed stratification period;0d, 45d, 90d, 135d, and 180d: seed stratification period; Em: embryo; En: endosperm; root, stem, and leaf as control tissues.

Eight genes were successfully performed in real-time PCR. Fig. 7 showed that *GA3ox4*, *GA20ox3*, *CYP707A4* and *KAO2* genes presented increased expression with dormancy release indicating these genes might be positively associated with seed dormancy release. *GA2ox* participated in the catabolism of biological active GA and *ZEP* involved in ABA biosynthesis indicating that these two genes might be negatively related with dormancy release[39,40]. In this study, the *GA2ox3* expression level Day 0 was almost the same as Day90 and Day180 The ZEP expression level also had no significantly difference among the samples. In this work three unigenes were annotated to the dormancy-associated protein gene (*DRM*) and the mRNA level of the *DRM1* gene Day90 and 180(Em) were significantly increased compared with the Day0 (*P*<0.05). This indicates that DRM activity may be correlated with morphological and physiological dormancy release in American ginseng seeds. GIGANTEA is one of the clock-associated protein that plays an important role in circadian oscillation and flowering-time regulation [41,42]. The expression level of *GAGANTEA2* was significantly higher in 180^th^ days’ endosperm than other samples (*P*<0.05). We also found that relative expression levels of *GA3ox4*, *GA20ox3*, *DRM1*, *ZEP* and *KAO2* were basically consistent with the semi-quantitative PCR showing lower levels in Day0 compared with other seed treatment samples.

## Conclusion

In this study, we obtained a comprehensive transcriptome of the seed dormancy release process of American ginseng using RNA-Seq technology. We assembled 78,207 unigenes and assigned 54,292 of them an explicit annotation. Many genes predicted to be involved in seed development and dormancy release were identified in this transcriptome. These findings substantially supplement existing sequence resources for American ginseng. Additionally, differentially expressed genes in the three stages of the seed dormancy release process (90DAS, 135DAS, 180DAS) were identified and functionally annotated with COG and KEGG database. These data will provide potential molecular targets for functional studies of genes in American ginseng seeds responding to development and dormancy release.

## Supporting Information

S1 Fig Quality evaluation and saturation analysis of DGE sequencing of American ginseng embryos at different physiological stratification stages. (TIF)

S2 Fig Distribution of genes’ coverage. (TIF)

S1 Table Primers used in the semi-quantitative PCR analysis of 30 selected unigenes. (XLSX)

S2 Table Selected unique transcripts that might be related with seed dormancy release or seed development. (XLSX)

S3 Table KEGG categories of non-redundant unigenes in the American ginseng seedslibraries. (PDF)

S4 Table Enriched GO annotations of differentially expressed genes during the American Ginseng seed dormancy release. (PDF)

S5 Table Enriched KEGG pathways of differentially expressed genes during seed dormancy release. (XLSX)

## References

[1] Thomas SC Li. Stratification of American ginseng seeds-problems and solutions. Native Plants Journal 2002; 3:109–111.

[2] Thomas SC Li Bedford KE, Sholberg PL. Improved germination of American ginseng seeds under controlled environments. HortTechnology 2000;10(1): 131–135.

[3] Chen Y.Technical manual of Chinese medicinal plant seed. People’s Medical Publishing House, Beijing, China; 1998.

[4] Baskin CC, Baskin JM. Seeds: ecology, biogeography, and evolution of dormancy and germination. Academic Press, San Diego; 1998.

[5] Li TSC. Asian and American ginseng-a review. HortTechnology 1995; 5:27–34.

[6] Li HQ, Huang SZ, Jing SW, Li JW, Jing DZ. Anatomical observation of the embryonic form of American ginseng. Special Wild Economic Animal and Plant Research 1995; 3:4–8.

[7] Li XZ, Qian SJ. Technology rule of American seed germination. Renshen Yan Jiu 2000; 12(4): 11–12.

[8] Chen SL, Sun YQ, Song JY, Li Y, Li CJ, et al Analysis of expressed sequence tags from Panax quniquefolium root. Yao Xue Xue Bao 2008; 43(6):657–663. 18822972

[9] Sun YZ, Niu YY, Li Y, Zhu YJ, Luo HM, Chen SL. Cloning and bioinformatic analysis of PqERF1 gene in Panx quinquefolius. Yao Xue Xue Bao 2011; 46(8):1008–1014. 22007529

[10] Sun C, Li Y, Wu Q, Luo HM, Sun Y, Song JY, et al De novo sequencing and analysis of the American ginseng root transcriptome using a GS FLX titanium platform to discover putative genes involved in ginsenoside biosynthesis. BMC Genomics 2010; 11: 262–279. 10.1186/1471-2164-11-262 20416102 PMC2873478

[11] Wu D, Austin RS, Zhou S, Brown D. The root transcriptome for North American ginseng assembled and profiled across seasonal development. BMC Genomics 2013; 14:564–577. 10.1186/1471-2164-14-564 23957709 PMC3751939

[12] Wu Q, Song JY, Sun Y, Suo FM, Li C, Luo HM, et al Transcript profiles of Panax quinquefolius from flower, leaf and root bring new insights into genes related to ginsenosides biosynthesis and transcriptional regulation. Physiol Plant 2010; 138(2):134–149. 10.1111/j.1399-3054.2009.01309.x 19947964

[13] Qi JJ, Zheng N, Zhang B, Sun P, Hu SN, Xu WJ, et al Mining genes involved in the stratification of Paris polyphylla seeds using high-throughput embryo transcritome sequencing. BMC Genomics 2013; 14: 358–373. 10.1186/1471-2164-14-358 23718911 PMC3679829

[14] Wang Z, Gerstein M, Snyder M. RNA-seq: a revolutionary tool for transcriptomics. Nat Rev Genet.2009; 10(1):57–63. 10.1038/nrg2484 19015660 PMC2949280

[15] Audic S, Claverie JM. The significance of digial gene expression profile. Genome Res. 1997; 7(10): 986–995. 9331369 10.1101/gr.7.10.986

[16] Liu G, Li W, Zheng P, Xu T, Chen L, Liu D, et al Transcriptomic analysis of Suli pear (Pyrus pyrifolia white pear group) buds during the dormancy by RNA-Seq. BMC Genomics 2012;13:700–717. 10.1186/1471-2164-13-700 23234335 PMC3562153

[17] Wang W, Wu Y, Messing J. RNA-Seq transcriptome analysis of Spirodela dormancy without reproduction. BMC Genomics 2014; 15(1): 60–72. 24456086 10.1186/1471-2164-15-60 PMC3933069

[18] Gao J, Yu X, Ma F, Li J. RNA-Seq analysis of transcriptome and blucosinolate metabolism in seeds and sprouts of Broccolo (Brassica oleracea var. italic). PLOS One 2014; 9(2): e88804 10.1371/journal.pone.0088804 24586398 PMC3937326

[19] Grabher MG, Haas BJ, Yassour M, Levin JZ, Thompson DA, Amit I, et al Full-length transcriptome assembly from RNA-Seq data without a reference genome. Nat Biotechnol 2011; 29(7): 644–52. 10.1038/nbt.1883 21572440 PMC3571712

[20] Iseli C, Jongeneel CV, Bucher P. ESTScan: a program for detecting, evaluating, and reconstructing potential coding regions in EST sequences. Proc Int Conf Intell Syst Mol Biol. 1999; 138–48. 10786296

[21] Conesa A, Gӧtz S, Garcia-Gόmez JM, Terol J, Talόn M, Robles M. Blast2GO: a universal tool for annotation, visualization and analysis in functional genomics research. Bioinformatics 2005; 21(18):3674–3676. 16081474 10.1093/bioinformatics/bti610

[22] Ye J, Fang I, Zheng H, Zhang Y, Chen J, et al WEGO: a web tool for plotting GO annotations. Nucleic Acids Res. 2006; 34(web server issue): W293–7. 16845012 10.1093/nar/gkl031 PMC1538768

[23] Meng XL, Liu M, Jiang KY, Wang BJ, Tian X, Sun SJ, et al De novo characterization of Japanese scallop Mizuhopecten yessoensis transcriptome and analysis of its gene expression following cadmium exposure. PLOS One 2013; 8(5) e64485 10.1371/journal.pone.0064485 23741332 PMC3669299

[24] Martazavi A, Williams BA, McCue K, Schaeffer L. and Wold B. Mapping and quantifying mammalian trnscriptomes by RNA-seq. Nat Methods 2008;5: 621–628. 10.1038/nmeth.1226 18516045 PMC13303166

[25] Livak KJ, Schmittgen TD. Analysis of relative gene expression data using real-time quantitative PCR and the 2^-ΔΔCT^ method. Methods 2001; 25: 402–408. 11846609 10.1006/meth.2001.1262

[26] Fei H, Tsang E, Cutler AJ. Gene expression during seed maturation in Brassica napus in relation to the induction of secondary dormancy. Genomics 2007; 89(3):419–428. 17207603 10.1016/j.ygeno.2006.11.008

[27] Hecht V, Vielle-Calzada JP, hartog MV, Schmidt ED, Boutilier K, Grossniklaus U, et al The Arabidopsis somatic embryogenesis receptor kinase1 gene is expressed in developing ovules and embryos and enhances embryogenic competence in culture. Plant Physiol. 2001; 127(3):803–816. 11706164 PMC129253

[28] Finkelsein R, Reeves W, Ariizumi T, Steber C. Molecular aspects of seed dormancy. Annu. Rev. Plant Bio. 2008; 59:387–415. 10.1146/annurev.arplant.59.032607.092740 18257711

[29] Li R, Yu C, Li Y, Lam T, Yiu S. SOAP2: An improved ultrafast tool for short read alignment. Bioinformatics. 2009; 25(15): 1966–1967. 10.1093/bioinformatics/btp336 19497933

[30] Hedley PE, Russell JR, Jorgensen L, Gordan S, Morris JA, Hackett CA, et al Candidated genes associated with bud dormancy release in blackcurrant (Ribes nigrum). BMC Plant Biol 2010; 10:202 10.1186/1471-2229-10-202 20840772 PMC2956551

[31] Finch-Savage WE, Cadman CS, Toorop PE, Lynm JR, Hilhorst HW. Seed dormancy release in Arobidopsis Cvi by dry after-ripening, low temperature, nitrate and light shows common quantitative patterns of gene expression direstec by environmentally specific sensing. Plant J. 2007; 51:60–78. 17461781 10.1111/j.1365-313X.2007.03118.x

[32] Yamauchi Y, Ogawa M, Kuwahara A, Hanada A, Kamiya Y, Yamaguchi S. Activation of gibberellins biosynthsis and response pathways by low temperature during imbibitions of Arabidopsis thaliana seeds. Plant Cell 2004; 16:367–378 14729916 10.1105/tpc.018143 PMC341910

[33] Cadman CS, Toorop PE, Hilhorst HW, Finch-Savage WE. Gene expression profiles of Arabidopsis Cvi seeds during dormancy cycling indicate a common underlying dormancy control mechanism. Plant J. 2006; 46:67–75. 10.1111/j.1365-313X.2006.02738.x 16709196

[34] Millar AA, Jacobsen JV, Ross JJ, Helliwell CA, Poole AT, Scofield G, et al Seed dormancy and ABA metabolism in Arabidopsis and barley: the role of ABA 8'-hydroxylase. Plant J. 2006; 45:942–954. 16507085 10.1111/j.1365-313X.2006.02659.x

[35] Lopez-Molina L, Mongrand S., McLachlin D., Chait BT. Chua NH. ABI5 acts downstream of ABI3 to execute an ABA-dependent growth arrest during germination. Plant J. 2002; 32:317–328. 12410810 10.1046/j.1365-313x.2002.01430.x

[36] Helliwell CA, Chandler PM, Poole A, Dennis ES, Peacock WJ. The CYP88A cytochrome P450, ent-kaurenoic oxidase, catalyzes three steps of the gibberellins biosynthesis pathway. PNAS 2001; 98(4):2065–2070. 11172076 10.1073/pnas.041588998 PMC29382

[37] Huq E, Tepperman JM, Quail PH. GIGANTEA is a nuclear protein involved in phytochrome signaling in Arabidopsis. PNAS 2000; 97(17):9789–9794. 10920210 10.1073/pnas.170283997 PMC16943

[38] Hedden P Thomas SG. Gibberellin biosynthesis and its regulation. Biochem. J. 2012; 444:11–25. 10.1042/BJ20120245 22533671

[39] Ross JJ, Reid JB, Swain SM, Hasan O, Poole AT, Hedden P, Willis CL. Genetic regulation of gibberellins deactivation in Pisum. Plant J 1995; 7:513–523

[40] Seiler C, Harshavardhan V T, Rajesh K, Reddy P S, Strickert M, Rolletschek H, et al ABA biosynthesis and degradation contributing to ABA homeostasis during barley seed development under control and terminal drought-stress conditions. Joural of Experimental Botany 2011; 62(8):2615–2632. 10.1093/jxb/erq446 21289079

[41] Park DH, Somers DE, Kim YS, Choy YH, Lim HK, Soh MS, et al Control of circadian rhythms and photoperiodic flowering by the Arabidopsis GIGANTEA gene. Science 1999; 285: 1579–1582. 10477524 10.1126/science.285.5433.1579

[42] Suarez-Lopez P, Wheatley K, Robson F, Onouchi H, Valverde F, Coupland G. CONSTANS mediates between the circadian clock and the control of flowering in Arabidopsis. Nature 2001; 410:1116–1120. 11323677 10.1038/35074138

