# Supplementary figures and images for: Transcriptomic Analysis of American Ginseng Seeds during the Dormancy Release Process by RNA-Seq
Source: PLoS One. 2015 Mar 19;10(3):e0118558. doi: 10.1371/journal.pone.0118558 (PMC4366157; doi:10.1371/journal.pone.0118558)

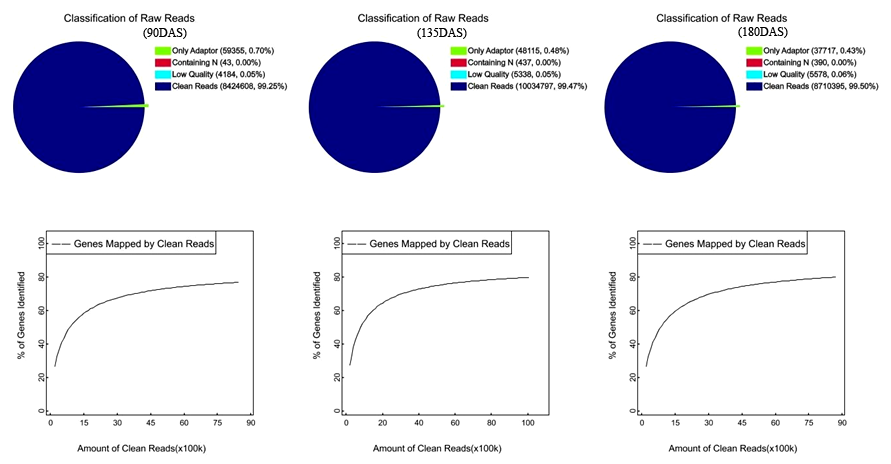

Supplement: S1 Fig — (TIF) [file pone.0118558.s001.tif]

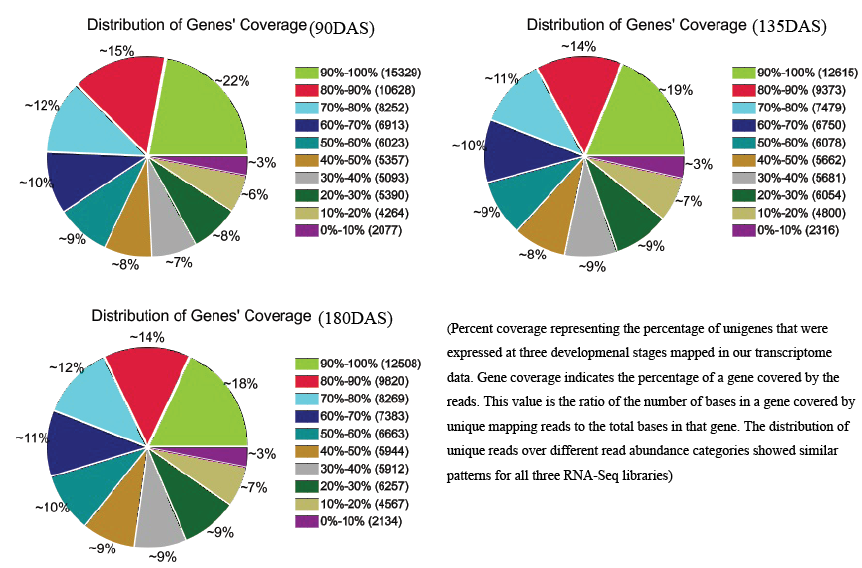

Supplement: S2 Fig — (TIF) [file pone.0118558.s002.tif]
