# Supplementary material for: Transcriptomic Analysis of American Ginseng Seeds during the Dormancy Release Process by RNA-Seq
Source: PLoS One. 2015 Mar 19;10(3):e0118558. doi: 10.1371/journal.pone.0118558 (PMC4366157; doi:10.1371/journal.pone.0118558)
Supplement: S3 Table — (PDF) [file pone.0118558.s005.pdf]

S3 Table. KEGG categories of non-redundant unigenes in the American ginseng seeds libraries

	#Pathway	Count (26909)	Pathway ID
1	Metabolic pathways	6117	ko01100
2	Biosynthesis of secondary metabolites	2633	ko01110
3	Plant hormone signal transduction	1536	ko04075
4	Plant-pathogen interaction	1464	ko04626
5	Spliceosome	1012	ko03040
6	Endocytosis	953	ko04144
7	RNA transport	919	ko03013
8	Glycerophospholipid metabolism	823	ko00564
9	Protein processing in endoplasmic reticulum	781	ko04141
10	Purine metabolism	706	ko00230
11	Ribosome	658	ko03010
12	Ether lipid metabolism	645	ko00565
13	Starch and sucrose metabolism	618	ko00500
14	mRNA surveillance pathway	573	ko03015
15	RNA degradation	566	ko03018
16	Ribosome biogenesis in eukaryotes	525	ko03008
17	Ubiquitin mediated proteolysis	519	ko04120
18	Pyrimidine metabolism	452	ko00240
19	Oxidative phosphorylation	419	ko00190
20	Phenylpropanoid biosynthesis	389	ko00940
21	Glycolysis / Gluconeogenesis	384	ko00010
22	Amino sugar and nucleotide sugar metabolism	360	ko00520
23	Phagosome	347	ko04145
24	Peroxisome	328	ko04146
25	Pyruvate metabolism	325	ko00620
26	Cysteine and methionine metabolism	283	ko00270
27	Aminoacyl-tRNA biosynthesis	262	ko00970
28	Circadian rhythm - plant	253	ko04712
29	Nucleotide excision repair	252	ko03420
30	ABC transporters	250	ko02010
31	Phosphatidylinositol signaling system	242	ko04070
32	Galactose metabolism	240	ko00052
33	Carbon fixation in photosynthetic organisms	240	ko00710
34	Inositol phosphate metabolism	229	ko00562
35	Homologous recombination	227	ko03440
36	Glutathione metabolism	227	ko00480
37	Basal transcription factors	223	ko03022
38	Stilbenoid, diarylheptanoid and gingerol biosynthesis	221	ko00945
39	Limonene and pinene degradation	219	ko00903
40	Citrate cycle (TCA cycle)	209	ko00020
41	Zeatin biosynthesis	205	ko00908
42	RNA polymerase	199	ko03020
43	Arginine and proline metabolism	193	ko00330
44	Fatty acid metabolism	189	ko00071

45	Biosynthesis of unsaturated fatty acids	189	ko01040
46	Fructose and mannose metabolism	188	ko00051
47	Pentose and glucuronate interconversions	186	ko00040
48	Glycerolipid metabolism	185	ko00561
49	alpha-Linolenic acid metabolism	184	ko00592
50	DNA replication	184	ko03030
51	Phenylalanine metabolism	184	ko00360
52	Flavonoid biosynthesis	175	ko00941
53	Cyanoamino acid metabolism	174	ko00460
54	Propanoate metabolism	161	ko00640
55	Mismatch repair	159	ko03430
56	Terpenoid backbone biosynthesis	158	ko00900
57	Pentose phosphate pathway	155	ko00030
58	Carotenoid biosynthesis	154	ko00906
59	Base excision repair	152	ko03410
60	Alanine, aspartate and glutamate metabolism	151	ko00250
61	Nitrogen metabolism	150	ko00910
62	Valine, leucine and isoleucine degradation	150	ko00280
63	N-Glycan biosynthesis	146	ko00510
64	Porphyrin and chlorophyll metabolism	145	ko00860
65	Proteasome	145	ko03050
66	Protein export	144	ko03060
67	Other glycan degradation	143	ko00511
68	Sphingolipid metabolism	143	ko00600
69	Ascorbate and aldarate metabolism	140	ko00053
70	Glycine, serine and threonine metabolism	140	ko00260
71	Glyoxylate and dicarboxylate metabolism	137	ko00630
72	Ubiquinone and other terpenoid-quinone biosynthesis	134	ko00130
73	Regulation of autophagy	133	ko04140
74	Glycosaminoglycan degradation	125	ko00531
75	Fatty acid biosynthesis	124	ko00061
76	Natural killer cell mediated cytotoxicity	118	ko04650
77	Valine, leucine and isoleucine biosynthesis	115	ko00290
78	Photosynthesis	112	ko00195
79	Tryptophan metabolism	112	ko00380
80	beta-Alanine metabolism	107	ko00410
81	SNARE interactions in vesicular transport	102	ko04130
82	Tyrosine metabolism	96	ko00350
83	Lysine degradation	94	ko00310
84	Selenocompound metabolism	92	ko00450
85	Sulfur metabolism	91	ko00920
86	Glycosphingolipid biosynthesis - ganglio series	86	ko00604
87	Glycosylphosphatidylinositol(GPI)-anchor biosynthesis	83	ko00563
88	Steroid biosynthesis	83	ko00100
89	Butanoate metabolism	82	ko00650
90	Phenylalanine, tyrosine and tryptophan	79	ko00400

	biosynthesis		
91	Diterpenoid biosynthesis	79	ko00904
92	Pantothenate and CoA biosynthesis	76	ko00770
93	Histidine metabolism	70	ko00340
94	Flavone and flavonol biosynthesis	64	ko00944
95	Nicotinate and nicotinamide metabolism	58	ko00760
96	Riboflavin metabolism	58	ko00740
97	Arachidonic acid metabolism	50	ko00590
98	Linoleic acid metabolism	49	ko00591
99	Lysine biosynthesis	48	ko00300
100	Folate biosynthesis	46	ko00790
101	Taurine and hypotaurine metabolism	45	ko00430
102	Benzoxazinoid biosynthesis	45	ko00402
103	One carbon pool by folate	44	ko00670
104	Circadian rhythm - mammal	43	ko04710
105	Glycosphingolipid biosynthesis - globo series	40	ko00603
106	Tropane, piperidine and pyridine alkaloid biosynthesis	39	ko00960
107	Isoquinoline alkaloid biosynthesis	39	ko00950
108	Non-homologous end-joining	37	ko03450
109	Photosynthesis - antenna proteins	35	ko00196
110	Monoterpenoid biosynthesis	34	ko00902
111	Vitamin B6 metabolism	34	ko00750
112	Glucosinolate biosynthesis	31	ko00966
113	Other types of O-glycan biosynthesis	30	ko00514
114	Sulfur relay system	29	ko04122
115	Brassinosteroid biosynthesis	26	ko00905
116	Thiamine metabolism	24	ko00730
117	C5-Branched dibasic acid metabolism	20	ko00660
118	Indole alkaloid biosynthesis	15	ko00901
119	Biotin metabolism	15	ko00780
120	Synthesis and degradation of ketone bodies	14	ko00072
121	Lipoic acid metabolism	12	ko00785
122	Anthocyanin biosynthesis	9	ko00942
123	Sesquiterpenoid biosynthesis	8	ko00909
124	Fatty acid elongation	6	ko00062
125	Caffeine metabolism	4	ko00232
126	Betalain biosynthesis	1	ko00965
