# Supplementary material for: Transcriptomic Analysis of American Ginseng Seeds during the Dormancy Release Process by RNA-Seq
Source: PLoS One. 2015 Mar 19;10(3):e0118558. doi: 10.1371/journal.pone.0118558 (PMC4366157; doi:10.1371/journal.pone.0118558)
Supplement: S4 Table — (PDF) [file pone.0118558.s006.pdf]

S4 Table. Enriched GO annotations for differentially expressed unigenes in American Ginseng seeds dormancy release. GO categories that were significantly enriched (p-value<0.05)were analyzed in pairwise comparisons (90DAS, 135DAS, and 180DAS)

Cellular component									
	90DAS-vs-135DAS			90DAS-VS-180DAS			135DAS-VS-180DAS		
Gene Ontology term	Cluster frequency		Crrected P-value	Cluster frequency		Corrected P-value	Cluster frequency		Corrected P-value
	Gene Number	out of 2757 genes (%)		Gene Number	out of 4553 genes (%)		Gene Number	Out of 1542 genes (%)	
Non-membrane-bounded organelle	264	9.6	2.25E-16						
Intracellular non-membrane-bounded organelle	264	9.6	2.25E-16						
Intracellular organelle part	595	21.6	2.56E-16						
Organelle part	674	24.2	1.72E-14	934	20.5	0.01433			
Ribonucleoprotein complex	180	6.5	2.00E-14						
External encapsulating structure	192	7	3.98E-13	282	6.2	8.86E-14	116	7.5	1.86E-09
Chromosomal part	73	2.6	2.24E-12				41	2.7	2.33E-06
Chromosome	81	2.9	2.27E-11				43	2.8	8.04E-05
Chromatin	43	1.6	2.29E-11	45	1	2.28E-05	28	1.8	2.85E-08
Macromolecular complex	445	16.1	1.25E-10						
Cell periphery	205	7.4	1.23E-09	315	6.9	5.55E-12	129	8.4	7.69E-09

Intracellular part	2183	79.2	1.29E-09			
Ribosome	91	3.3	2.03E-09			
Intracellular	2192	79.5	2.46E-09			
Cell wall	99	3.6	1.64E-07	139	3.1	1.70E-06
Cytoplasm	1069	38.8	3.11E-07	1652	36.3	0.00477
Cytoplasmic part	1067	38.7	4.10E-07	1648	36.2	0.0069
Ribosomal subunit	60	2.2	1.37E-05			
Mitochondrial inner membrane	43	1.6	2.49E-05	53	1.2	0.00583
Mitochondrial membrane part	36	1.3	2.84E-05	44	1	0.0036
Intracellular organelle	1961	71.1	3.66E-05			
Organelle membrane	267	9.7	6.40E-05			
Mitochondrial respiratory chain	31	1.1	8.97E-05	40	0.9	0.00076
Respiratory chain	31	1.1	8.97E-05	40	0.9	0.00076
Mitochondrial membrane	51	1.8	1.30E-04			
Anchored to membrane	34	1.2	1.40E-04			
Mitochondrial part	69	2.5	1.90E-04			
Organelle	1974	71.6	2.10E-04			
Mitochondrion	69	2.5	6.40E-04			
Membrane-enclosed lumen	117	4.2	6.40E-04			
Mitochondrial envelope	53	1.9	9.50E-04			
Nuclear lumen	114	4.1	1.21E-03			
Organelle lumen	114	4.1	1.21E-03			

Intracellular organelle lume	114	4.1	1.21E-03						
Organelle inner membrane	86	3.1	2.87E-03						
Extracellular region	85	3.1	3.69E-03	144	3.2	2.32E-07	67	4.3	1.08E-07
Organelle envelope	203	7.4	4.14E-03	309	6.8	0.01766			
Proton-transporting V-type ATPase complex	12	0.4	1.16E-02	20	0.4	3.35E-06			
Plastid	521	18.9	1.69E-02	844	18.5	0.00141			
Envelope	203	7.4	2.12E-02	314	6.9	3.67E-02			
Plastid part	240	8.7	2.41E-02	390	8.6	0.00053			
Proton-transporting two-sector ATPase complex	25	0.9	2.47E-02	38	0.8	0.00149			
intracellular Membrane-bounded organelle	1673	60.7	3.68E-02						
NADH dehydrogenase complex	16	0.6	7.68E-02						
Thylakoid				158	3.5	0.00627			
Chloroplast thylakoid				137	3	0.00819			
Organelle subcompartment				145	3.2	0.00926			
Plastid thylakoid				143	3.1	0.01491			
Vacuolar proton-transporting V-type ATPase complex				8	0.2	0.02432			
vacuolar membrane				10	0.2	0.0456			
Photosystem							18	1.2	0.00066

Photosynthetic membrane							21	1.4	0.00324
External encapsulating structure part							6	0.4	0.01933
Thylakoid							25	1.6	0.02301
Chloroplast							65	1.6	0.04047
**Molecular Function**									
	90DAS-VS-135DAS			90DAS-VS-180DAS			135DAS-VS-180DAS		
**Gene Ontology term**	**Cluster frequency**		**Corrected P-value**	**Cluster frequency**		**Corrected P-value**	**Cluster frequency**		**Corrected P-value**
	**gene number**	**out of 3049 genes (%)**		**gene number**	**out of 5295 genes (%)**		**gene number**	**out of 1842 genes (%)**	
Structural molecule activity	125	4.1	6.27E-12	48	0.9	1.43E-03			
Oxidoreductase activity, acting ont the aldehyde or oxo group of donors, NAD or NADP as acceptor	33	1.1	0.00194						
S-methyltransferase activity	11	0.4	0.00404						
Oxidoreductase activity, acting on the aldehyde or oxo group of donors	39	1.3	0.01966						
Water transmembrane	11	0.4	0.02348						

transporter activity									
cis-trans isomerase activity	23	0.8	0.03899						
Guanyl ribonucleotide binding	82	2.7	0.04476	133	2.5	0.00202			
Iron ion binding				61	1.2	0.00275	70	3.8	2.35E-07
oxidoreductase Activity, acting on the aldehyde or oxo group of donors				18	0.3	0.0038			
Heme-copper terminal oxidase activity				15	0.3	0.00653			
O-methyltransferase activity				12	0.2	0.00837			
Efflux transmembrane transporter activity				23	0.4	0.0238			
Oxidoreductase activity, acting on the CH-CH group of donors, oxygen as acceptor				94	1.8	0.01034	13	0.7	0.01125
Oxidoreductase activity, acting on the Ch-OH group of donors, NAD or NADP as acceptor				119	2.2	0.04729	83	4.5	0.00023
Oxidoreductase activity, acting on CH-OH group of donors				776	14.7	4.05E-10			
Oxidoreductase activity							343	18.6	1.58E-16
Amine transmembrane transporter activity							31	1.7	1.03E-09

Carboxylic acid transmembrane transporter activity							18	1	5.32E-07
Amino acid transmebrane transporter activity							16	0.9	6.29E-07
Organic acid transmembrane transporter activity							23	1.2	4.11E-06
Oxidoreductase activity, acting on the Ch-CH group of donors							98	5.3	7.44E-06
Oxidoreductase activity, acting on paired donors, with oncorporation or reduction of molecular oxygen							48	2.6	2.97E-03
Active transmembrane transporter activity							103	5.6	4.29E-03
Antioxidant activity							30	1.6	4.81E-03
Carboxylesterase activity							32	1.7	2.90E-02
Monooxygenase activity							38	2.1	3.09E-02
Monosaccharide transmembrane transporter activity							7	0.4	4.88E-02
**Biological Process**									
	90DAS-VS-135DAS			90DAS-VS-180DAS			135DAS-VS-180DAS		

Gene Ontology term	Cluster frequency		Corrected P-value	Cluster frequency		Corrected P-value	Cluster frequency		Corrected P-value
	gene number	Out of 2754 genes (%)		Gene Number	Out of 4607 genes (%)		Gene number	Out of 1642 genes (%)	
Nucleosome organization	36	1.3	2.26E-09						
Cellular macromolecular complex subunit organization	65	2.4	7.29E-05						
Macromolecular complex subunit organization	74	2.7	0.00018						
DNA methylation on cytosine	20	0.7	0.0004						
DNA methylation	22	0.8	0.00048						
Hydrogen transport	36	1.3	0.00124	57	1.2	1.53E-06			
Chromosome organization	91	511	0.00137						
Response to inorganic substance	144	5.2	0.00329						
Response to metal ion	126	4.6	0.0046						
Chromatin organization	80	2.9	0.01031						
Regulation of photosynthesis, light reaction	6	0.2	0.01048						
Regulation of	6	0.2	0.01048						

generation of precursor metabolites and energy									
Response to red or far red light	52	1.9	0.01228						
Response to stimulus				1285	27.9	3.35E-07	525	32	1.58E-10
Organic substance metabolic				28	0.6	1.36E-06	13	0.8	0.008
Carbon fixztion				23	0.5	1.96E-06	12	0.7	0.00124
Response to abiotic stimulus				439	9.5	7.20E-04			
Response to chemical stimulus				600	13	4.19E-03			
Response to stress				652	14.2	5.54E-03	294	17.9	1.82E-09
Proton transport				34	0.7	1.03E-02			
Aromatic compound biosynthetic process				60	1.3	1.04E-02			
Response to auxin stimulus				26	0.6	1.33E-02			
Nucleosome organization				34	0.7	2.33E-02	24	1.5	2.86E-06
Nitrogen compound transport							37	2.3	4.08E-10
Amine transport							36	2.2	6.66E-10
Organic substance transport							72	4.4	1.30E-09
Organic acid transport							30	1.8	1.48E-07
Carboxylic acid							30	1.8	1.48E-07

transport						
Amino acid transport				27	1.6	1.63E-07
Anion transport				36	2.2	2.92E-05
Organic anion transport				14	0.9	3.89E-05
Aromatic amino acid transport				6	0.4	3.90E-04
Cell wall organization				33	2	9.40E-04
Protein folding				11	0.7	8.83E-03
Cell communication				35	2.1	1.75E-02
DNA methylation				14	0.9	3.94E-02
Aminoglycan catabolic process				5	0.3	4.43E-02
Hydrogen peroxide metabolic process				12	0.7	4.18E-02
